# Nephrotic-range Albuminuria as the presenting symptom of Dent-2 disease

**DOI:** 10.1186/s13052-015-0152-4

**Published:** 2015-06-25

**Authors:** Chiara De Mutiis, Andrea Pasini, Claudio La Scola, Fabrizio Pugliese, Giovanni Montini

**Affiliations:** Nephrology and Dialysis Unit, Department of Pediatrics, Azienda Ospedaliero-Universitaria Sant’Orsola-Malpighi Bologna, Via Massarenti 11, 40138 Bologna, Italy

**Keywords:** Dent disease, Low molecular weight proteinuria (LMWP), Hypercalciuria, *OCRL*, *CLCN5*, p.Arg318Cys

## Abstract

Dent disease is a rare X-linked tubulopathy with low molecular weight proteinuria, hypercalciuria, nephrolithiasis, nephrocalcinosis and progressive renal failure. We describe the case of a 9-year-old boy who presented with nephrotic-range albuminuria at the age of 3 years. In the absence of a clear diagnosis, a renal biopsy was performed at 4 years, which revealed minimal change disease. Due to the presence of low molecular weight proteinuria, even in the absence of hypercalciuria, a diagnosis of Dent disease was considered. While there were no mutations in the *CLCN5 gene*, the diagnosis was confirmed by the presence of a missense mutation (p.Arg318Cys) in the *OCRL* gene. Conclusion: Given the large phenotypic variability of the disease and based on our experience, we believe that children with low molecular weight proteinuria, even without hypercalciuria, should be investigated for Dent disease.

## Background

Dent disease is a rare, X-linked disorder characterized by low molecular weight proteinuria (LMWP), hypercalciuria, and at least one of the following criteria: nephrolithiasis, nephrocalcinosis, hematuria, hypophosphatemia or progressive renal failure [1]. Other features of proximal tubulopathy can be present, such as glycosuria, aminoaciduria and acidification defects [1, 2]. It is caused by mutations in the *CLCN5* gene (Dent-1 disease, OMIM #300009) in ~ 60 % of cases, in the *OCRL* gene (Dent-2 disease, OMIM#300555) in ~15 % of cases,, while in the remaining 25 % of cases [3, 4] other unidentified genes are responsible for the disease. The incidence and prevalence of Dent-1 and 2 disease are unknown. Several recent papers have reported an atypical phenotype of Dent-1 disease, presenting with high range proteinuria and LMWP as a unique clinical feature [5–8].

We describe a boy who presented with asymptomatic high range proteinuria, LMWP and normocalciuria at the age of 3 years, and who was subsequently diagnosed with Dent-2 disease with an *OCRL* mutation.

## Case report

At the age of 10 months, the child presented with proteinuria and hematuria (urine dipstick: pH 6, S.G. 1020, Hb 1+, Albumin 3+) following a febrile viral infection. Renal function, C3 and C4 serum levels were all normal; extractible nuclear antigen, antinuclear antibodies and anti DNA antibodies were negative. Proteinuria (600–800 mg/day) was confirmed in subsequent evaluations without hypoalbuminemia, edema or hyperlipidemia. Kidney ultrasound was normal. There was no family history of kidney disease.

At the age of 4, he was referred to our hospital with nephrotic range proteinuria (uPr/uCr mg/mg between 1.4 and 4.4), albuminuria 100–300 mg/dl and a high component of LMWP, but without hypercalciuria (uCa/uCr mg/mg: 0.38 normal < 0.4 for children from 3 to 5 years), nephrocalcinosis or signs of tubular damage (tubular reabsorption of phosphate > 82 %, absence of glycosuria and aminoaciduria). A technetium-99 m-dimercaptosuccinate (DMSA) scan revealed: “poor renal accumulation of radionuclide 3 h after injection”. A renal biopsy was then performed which showed “minor glomerular abnormalities” typical of minimal change disease.

The child underwent regular six-monthly check-ups during which time no clinical, laboratory or renal US changes were observed. Albuminuria, LMWP were persistently elevated while values of urinary calcium were within the normal range for age.

On the basis of some reports of patients with Dent-1 disease, whose initial clinical presentation was proteinuria with LMWP but without hypercalciuria [5, 6], we decided to screen our patient for CLCN5 and OCRL1 mutations. A missense mutation (p.Arg318Cys) was identified in exon 11 of the *OCRL* gene. Carrier status was confirmed in the mother.

At present, the child is nine years old and has normal renal function (eGFR: 130 ml/min/1.73 m^2^), proteinuria (uPr/uCr: 2.2) with albuminuria 201 mg/L, alpha-1 microglobulin 329 mg/L (normal 0–12 mg/L), and beta-2 microglobulin 128 mg/L (normal < 10.0 mg/L), without hypercalciuria (uCa/uCr: 0.24 normal < 0.25) [9, 10], nephrolithiasis or nephrocalcinosis. Mild elevations of muscle enzymes (CPK 332 U/L, LDH 375 U/L) have been documented, without clinical evidence of muscle weakness. Growth, intellectual development and ophthalmic examinations have all been normal up to now.

## Discussion

We report a child with atypical Dent-2 disease, documented by an *OCRL* mutation, who presented with isolated proteinuria in the absence of any of the other classic criteria, particularly hypercalciuria. To date, approximately 250 families with Dent disease have been reported [1], of whom around 40 patients had Dent-2 disease, with various *OCRL* mutations described [1, 4].

Some studies have shown a large variability in the clinical phenotype of Dent disease, so much so that we can consider this disease to be a heterogeneous group of proximal tubular defects called “The Dent disease complex” [11]. A proportion of them have glomerular involvement, as was demonstrated in six of the nine children with Dent-1 disease presenting with asymptomatic high range proteinuria associated with LMWP, without hypercalciuria, and showing a focal segmental glomerulosclerosis and/or focal global glomerulosclerosis on biopsy [5–7]. Ludwig et al described two patients with LMWP as the only symptom of Dent-1 disease [8]. Cramer MT et al recently reported seven males with nephrotic-range proteinuria and *CLCN5* mutations, two of which showed no hypercalciuria (Table 1). Our patient had a very similar clinical presentation to these patients, with the exception of minimal change disease on biopsy, which, however, was performed early in the course of the disease, perhaps before the development of sclerotic lesions.

**Table 1:** Patients with Dent-1 disease in the absence of hypercalciuria reported in the literature

Parameters		Patient 1 [5]	Patient 2 [5]	Patient 3 [5]	Patient 4 [5]	Patient 5 [6]	Patient 6 [7]	Patient 7 [7]	Patient 8 [8]	Patient 9 [8]
Age (years)		9	11	14	9	9	6	6	36	15
CrCl (cc/min/1.73 m2)	>90	97	105	95.5	126	79	98	135	>80	>80
S-Alb (gr/dl)	3.5-5.3	4.5	4.9	4.1	4.4	4.6	4.5	4.9	-	-
U-prot (mg/day)	<200	800–1000	600–1400	750–1100	1240	1321	–	-	-	-
U-prot/U-Cr (mg/mg)	<0.2	-	-	-	-	-	3.4	2.6	-	-
U-β2MG (mg/l)	< 10	91.2	103	120	74.4	52.7	-	-	>10	>10
U-β2MG (mcg/g cr)	< 132						166.1		>132	>132
U-Ca/U-Cr (mg/mg)	< 0.5 for 1-3y	0.15	0.14	0.13	0.12	0.07	0.18	0.10	< 0.25	< 0.25
	<0.4 for 3-5 y									
	<0.3 for 5-7 y									
	< 0.25 for >7y									
U-gluc	absent	neg	neg	neg	neg	-	-	-	-	-
TRP (%)	82-95	87.4	91.8	86.2	95.8		85	-	-	-
Nephrocalcinosis by Renal US		Yes	Yes	No	No	No	No	Yes	No	No

*CrCl* creatinine clearance, *S-Alb* serum albumin, *U-prot* urinary protein, *U-β2MG* urinary beta2microglobuline, *U-Ca/U-Cr* urinary calcium/urinary creatinine, *U-prot/U-Cr* urinary protein/urinary creatinine, *U-gluc* urinary glucose, *TRP* tubular reabsorption of phosphate, *Renal US* renal ultrasound

In this respect, it is possible that these children could benefit from ACE inhibition, which may help protect the kidneys from the appearance of sclerotic lesions secondary to proteinuria. Copelovitch et al. [6] documented moderate improvement in proteinuria in a Dent patient after starting enalapril therapy, However, only a small number of the reported patients have been treated with these drugs [5, 7]. More studies are needed in order to draw more solid conclusions.

Our patient has a missense mutation (p.Arg318Cys) in exon 11 of the *OCRL* gene. To our knowledge, only 6 patients with the same mutation and Dent disease have been described: two brothers by Hoopes et al. [3], one male by Sekine T. et al [9], two brothers by Böckenhauer et al. [4] and one male by Hichri et al [12] (Table 2). Interestingly, the same mutation was identified by Hichri et al in another patient diagnosed with Lowe syndrome. The patients described by Hoopes and Böckenhauer had carrier females, as did our child, while the boy described by Sekine likely had a de novo mutation.

**Table 2:** Patients with Dent-2 disease with the same missense mutation in exon 11 (p.Arg318Cys) as our patient

	Patient 1 [3]^a^	Patient 2 [3]^a^	Patient 3 [9]	Patient 4 [4]^b^	Patient 5 [4]^b^	Patient 6 [12]	Our patient
Age at presentation	-	-	7	-	-	-	2
Age at diagnosis (years)	22	27	15	12	17	7	6
CrCl (cc/min/1.73 m2)	-	-	90	107	77	-	140
Hypercalciuria (>0.24)	yes	yes	no	yes	yes	-	no
LMWP	yes	yes	yes	yes	yes	yes	yes
Low TRP (%)	-	-	-	no	no	-	no
Renal biopsy	-	-	minor glomerular abnormalities	-	-	-	minimal change disease
Elevated CPK/LDH	no/no	no/no	yes/no	yes/yes	yes/yes	-	yes/yes
glycosuria	no	no	no	no	no	-	no
aminoaciduria	-	-	no	no	no	-	no
Renal tubular acidosis	-	-	no	no	no	-	no
Nephrocalcinosis/stones	no/no	no/no	no/no	yes/no	yes/no	-	no/no
Ocular involvement	no	no	no	no	no	-	no
Cognitive function	mild mental retardation	mild mental retardation	normal	normal	normal	normal	normal
Growth	-	-	normal	normal	short stature	-	normal

*CrCl* creatinine clearance, *LMWP* low molecular weight proteinuria, *TRP* tubular reabsorption of phosphate, *CPK* creatine phosphate kinase, *LDH* lactate dehydrogenase

^a^,^b^brothers

At diagnosis, the six patients had a mean age of 16.6 years and showed the classic hallmarks of Dent disease with LMWP and hypercalciuria, except for the patient described by Sekine, who lacked hypercalciuria and the patient reported by Hichri, whose data is not available. Two patients (3 and 5 in Table 2) developed a reduction in GFR during adolescence, while a renal biopsy performed only in patient 3, demonstrated minimal change disease, as was found in our patient. This histologic finding could lead to misdiagnosis and inappropriate and ineffective therapies such as the use of corticosteroids or immunosuppressive agents.

Furthermore, patients with Dent- 2 disease may share some extra-renal symptoms with the more severe Lowe’s syndrome, such as peripheral cataracts, mental impairment, growth retardation and elevated creatinine kinase and lactate dehydrogenase, without evidence of muscle weakness [4, 10]. Regarding the p.Arg318Cys mutation, the patient with Lowe syndrome reported by Hichri et al [12] presented with severe myopia, while patients with Dent disease lack cataracts or other forms of ocular damage. An increase of CPK and LDH enzymes was present in our patient and in the two brothers described by Böckenhauer (patients 4 and 5 in Table 2). Some degree of mental impairment was detected in patients 1 and 2 and short stature was noted in patient 5. These figures confirm that, even within the same genotype, there is a certain variability in the expression of the severity of the disease.

The absence of hypercalciuria in our patient may be due to his younger age and it is possible that he could express all the hallmarks of Dent disease in the future, but there is also the possibility that other mechanisms could regulate and compensate for the tendency to increase calcium excretion, reducing the degree of calciuria.

Therefore, the presence of hypercalciuria, typically considered pathognomonic of Dent disease, could be absent and this should be considered as part of the variability in the phenotype of this disease.

## Conclusion

Dent-2 disease may have a variable phenotype, lacking all the classic hallmarks, as described for some patients with *CLCN5* mutations [5–8], such that it could remain underdiagnosed.

Based on our experience and data from the literature, the diagnosis of Dent disease should be considered in all patients with high range proteinuria and LMWP, even in the absence of hypercalciuria. The *CLCN5* gene, and if negative, the *OCRL1* gene should be analyzed before considering a renal biopsy in males with nephrotic range proteinuria and no clinical signs of nephrotic syndrome. Extra-renal symptoms (ocular involvement, mental impairment, growth retardation and elevated creatinine kinase and lactate dehydrogenase) should be looked for and could lead to a diagnosis of Dent-2 disease.

## Consent

Written informed consent was obtained from the patient for publication of this Case report and any accompanying images. A copy of the written consent is available for review by the Editor-in-Chief of this journal.

## Abbreviations

CrCl: Creatinine clearance
LMWP: Low molecular weight proteinuria
TRP: Tubular reabsorption of phosphate
CPK: Creatine phosphate kinase
LDH: Lactate dehydrogenase
S-Alb: Serum albumin
U-prot: Urinary protein
U-β2MG: Urinary beta2microglobuline
U-Ca/U-Cr: Urinary calcium/urinary creatinine
U-prot/U-Cr: Urinary protein/urinary creatinine
U-gluc: Urinary glucose
Renal US: Renal ultrasound
ACE: Angiotensinconverting enzyme
